# Untargeted metabolomics reveals dynamic changes in metabolic profiles of rat supraspinatus tendon at three different time points after diabetes induction

**DOI:** 10.3389/fendo.2023.1292103

**Published:** 2023-11-20

**Authors:** Kuishuai Xu, Liang Zhang, Tianrui Wang, Zhongkai Ren, Tengbo Yu, Yingze Zhang, Xia Zhao

**Affiliations:** ^1^ Department of Sports Medicine, Affiliated Hospital of Qingdao University, Qingdao, China; ^2^ Department of Sports Medicine, Qingdao Municipal Hospital, Qingdao, Shandong, China

**Keywords:** diabetes mellitus, rotator cuff, supraspinatus, metabolomics, biomarker discovery, progression

## Abstract

**Objective:**

To investigate the dynamic changes of metabolite composition in rat supraspinatus tendons at different stages of diabetes by untargeted metabolomics analysis.

**Methods:**

A total of 80 Sprague–Dawley rats were randomly divided into normal (NG, n = 20) and type 2 diabetes mellitus groups (T2DM, n = 60) and subdivided into three groups according to the duration of diabetes: T2DM-4w, T2DM-12w, and T2DM-24w groups; the duration was calculated from the time point of T2DM rat model establishment. The three comparison groups were set up in this study, T2DM-4w group *vs*. NG, T2DM-12w group *vs*. T2DM-4w group, and T2DM-24w group *vs*. T2DM-12w group. The metabolite profiles of supraspinatus tendon were obtained using tandem mass spectrometry. Metabolomics multivariate statistics were used for metabolic data analysis and differential metabolite (DEM) determination. The intersection of the three comparison groups’ DEMs was defined as key metabolites that changed consistently in the supraspinatus tendon after diabetes induction; then, Kyoto Encyclopedia of Genes and Genomes (KEGG) pathway enrichment analysis was performed.

**Results:**

T2DM-4w group vs. NG, T2DM-12w group vs. T2DM-4w group, and T2DM-24w group vs. T2DM-12w group detected 94 (86 up-regulated and 8 down-regulated), 36 (13 up-regulated and 23 down-regulated) and 86 (24 up-regulated and 62 down-regulated) DEMs, respectively. Seven key metabolites of sustained changes in the supraspinatus tendon following induction of diabetes include D-Lactic acid, xanthine, O-acetyl-L-carnitine, isoleucylproline, propoxycarbazone, uric acid, and cytidine, which are the first identified biomarkers of the supraspinatus tendon as it progresses through the course of diabetes. The results of KEGG pathway enrichment analysis showed that the main pathway of supraspinatus metabolism affected by diabetes (p < 0.05) was purine metabolism. The results of the KEGG metabolic pathway *vs*. DEMs correlation network graph revealed that uric acid and xanthine play a role in more metabolic pathways.

**Conclusion:**

Untargeted metabolomics revealed the dynamic changes of metabolite composition in rat supraspinatus tendons at different stages of diabetes, and the newly discovered seven metabolites, especially uric acid and xanthine, may provide novel research to elucidate the mechanism of diabetes-induced tendinopathy.

## 1 Introduction

Tendinopathy is one of the common diseases of the human motor system, and many factors, including aging, inflammation, chronic injury, and metabolic diseases, lead to tendinopathy (1). Among these, diabetes mellitus is a major factor affecting tendon quality and leading to tendinopathy (2). In recent years, the increasing number of people with diabetes has prompted a large number of studies (3, 4) to focus on the adverse effects of diabetes on tendons. Some studies have shown that diabetes alters the physical and chemical properties of tendons and the arrangement of collagen fibers, extracellular matrix composition, and biomechanics in a high-glucose microenvironment (5). The current study demonstrated that the degree of tendinopathy gradually worsens and the biomechanical properties decrease in the supraspinatus tendon of rats at 2, 4, 8, and 12 weeks after diabetes induction (6). However, studies on how diabetes leads to the continuous progression and deterioration of rotator cuff tendinopathy have not been reported; also, the changes in endogenous small molecules and the involved biological pathways with the progression of diabetes remain unclear. With the increasing development of metabolomics, metabolomic-based studies provide novel ideas for the study of tendinopathy. In a recent non-targeted metabolomics study, Sikes et al. (7) demonstrated that creatine, inositol, and lipid signaling pathways may be involved in the development of tendinopathy in mouse models. Although the subjects of this study were not animal models of diabetes, untargeted metabolomics results showed subtle differences between samples providing new insights into the mechanisms of disease development and progression.

In previous studies, metabolomics techniques have played a significant role in investigating the mechanisms by which diabetes leads to the continuous progression and deterioration of diseases in the kidney (8), microvasculature (9), and retina (10), as well as obesity (11). Peng et al. (8) found that two differential metabolites (DEMs) (Asn-Met-Cys-Ser and Asn-Cys-Pro-Pro) increased with the progression of proteinuria in diabetic nephropathy, and the newly discovered serum metabolites could be used as biomarkers for the continuous progression of diabetic nephropathy. Li et al. (9) identified 24 metabolites that reflect the metabolic changes at different stages of diabetic peripheral vascular disease by untargeted metabolomics. Yun et al. (10) used targeted metabolomics and showed that the mechanism by which diabetes leads to the development and progression of retinopathy might be related to carnitine and phosphatidylcholine. Lv et al. (11) identified DEMs using metabolomics, which might be putative biomarkers for assessing pancreatic β-cell function at different stages of diabetes. Although several studies have investigated the mechanism by which diabetes leads to the development and progression of various complications using metabolomics techniques, the dynamic changes in the metabolic profiles of supraspinatus tendons at different stages after diabetes induction have not been reported.

**Figure 1:**
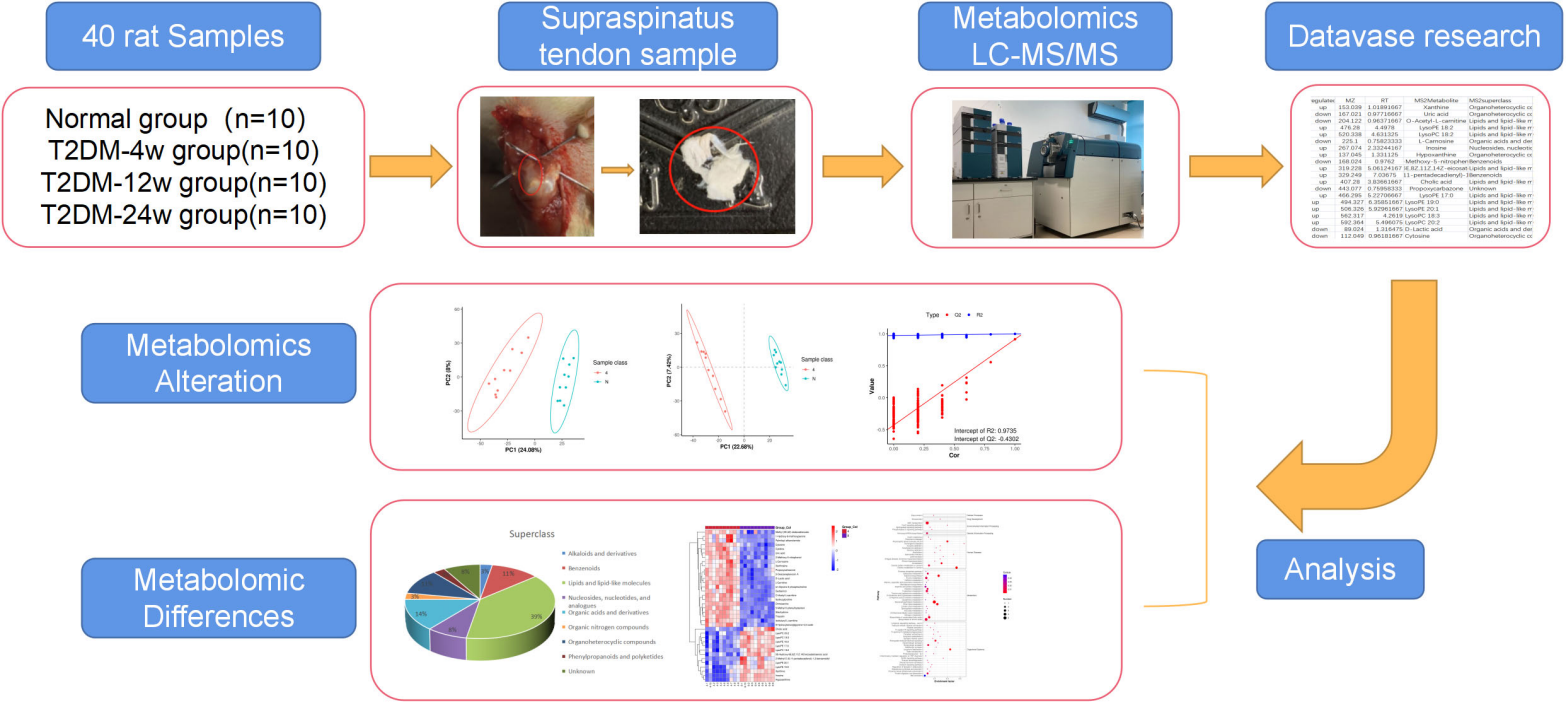
Study design and metabolomic analysis of the supraspinatus tendon in diabetic rats. Overview of the cohort (including 10 normal samples, 10 type 2 diabetes 4-week samples, 10 type 2 diabetes 12-week samples, and 10 type 2 diabetes 24-week samples) and study design (including metabolomics LC-MS/MS, database search, detection, identification, and quantitative analysis of metabolites, and screening of DEMS).

Patients/animal tendons with different duration of diabetes have diverse molecular profiles of metabolites. Consequently, 40 supraspinatus tendon samples were extracted from normal and diabetic rats in this study to reveal a list of metabolites associated with the progression of diabetic tendinopathy (**Figure 1**). Next, we performed untargeted metabolomics testing on all samples, screened for DEMs that consistently changed throughout diabetes, and performed hierarchical cluster analysis and KEGG pathway analysis. We speculated that this study provides new ideas for exploring the mechanisms by which diabetes leads to the development and progression of rotator cuff tendinopathy.

## 2 Materials and methods

### 2.1 Experimental grouping and establishment of type 2 diabetic rat model

A total of 80 Sprague–Dawley rats (6-week-old, 200–220 g, Beijing Vital River, production license No. SCXK (Zhejiang) 2019-0001) were divided into four groups according to random number table method: normal, diabetic 4-week, diabetic 12-week, and diabetic 24-week group. The normal group was fed a normal diet, and the diabetic group was fed a high-sugar and high-fat diet (Mediscience, MD12033) for 4 weeks. subsequently, diabetic rats were injected 40 mg/kg streptozotocin (STZ, Aladdin) intraperitoneally on an empty stomach (12), and rats fed a normal diet were injected with an equivalent volume of sodium citrate buffer (SSC, BIOISCO, 40 mg/kg). Three days after the injection of STZ solution, fasting blood glucose (FBG) concentration was measured in the rat tail tip blood; if the blood glucose level was ≥ 16.7 mmol/L for 3 consecutive days, the model was considered successful (13). Rats whose blood glucose did not reach the criterion were excluded and quantitatively supplemented.

### 2.2 Metabolite sample preparation

The bilateral supraspinatus tendons of rats were removed, the excess muscle components around the tendons were separated, the tendons were immediately placed in a precooled phosphate-buffered saline (PBS), the stains and blood on the tissue surface were washed, the liquid on the surface was blotted, and the treated tissues were rapidly placed in precooled numbered enzyme-resistant −192°C ultra-low temperature-threaded mouth cryogenic vials without enzymes, snap frozen in liquid nitrogen for 3-4 h, and placed in a −80°C freezer. The samples were stored on dry ice during shipment for metabolomics analysis. Moreover, metabolomic analysis requires at least 50 mg of tissue, and supraspinatus tendon tissue from both rotator cuffs is undermass in one animal; hence, supraspinatus tendon tissue from two animals (four shoulders) needs to be pooled to construct one metabolomic sample.

### 2.3 Untargeted metabolomic analysis of supraspinatus tendon

Metabolites were extracted from tendons with 50% methanol buffer. The processes of sample collection, storage, and preparation were consistent with that of Han et al. (14). Pooled quality control (QC) samples were prepared by mixing 10 μL of each extraction mixture. This analysis was carried out on an ultra-performance liquid chromatography (UPLC)–MS/MS system: a UPLC (UltiMate 3000 HPLC, Thermo Fisher Scientific, San Jose, CA, USA) connected to a high-resolution tandem mass spectrometer (Q-Exactive, Thermo Fisher Scientific, Saint Louis, MO, USA). The supernatants were collected and used for metabolomic analysis. LC/MS and untargeted metabolomics raw data were analyzed at LC-Bio Technology Co., Ltd (Hangzhou, Zhejiang Province, China). The instrument parameters were set using previously reported methods (14). A high-resolution tandem mass spectrometer Q-Exactive (Thermo Scientific) was used to detect metabolites eluted form the column. The Q-Exactive was operated in both positive and negative ion modes. Precursor spectra (70–1050 m/z) were collected at 70,000 resolution to hit an AGC target of 3e6. The maximum inject time was set to 100 ms. A top 3 configuration to acquire data was set in DDA mode. Fragment spectra were collected at 17,500 resolution to hit an AGC target of 1e5 with a maximum inject time of 80 ms. In order to evaluate the stability of the LC-MS during the whole acquisition, a quality control sample (Pool of all samples) was acquired after every 10 samples.

Metabolomics datasets were analyzed using the open-source software metaX, and univariate and multivariate analyses were performed to obtain DEMs between the three comparison groups. Collecting, identifying, and analyzing baseline data was similar to that reported in a recent study by Yang et al. (15). The p-value was adjusted by Benjamini–Hochberg’s approach. Variable Importance in Projection (VIP) value > 1, FC > 2 or < 0.5, and an adjusted p-value < 0.05 were selected as DE features and used for further analyses. These criteria were selected according to those described previously (16). The metabolites included in the intersection of DEMs between the three comparison groups were identified by Venn Diagram. The relative content of the metabolites was calculated by a Z-score plot, and then the trend change of metabolites between the three diabetes stages was analyzed. All DEM features/metabolites were annotated in KEGG (http://www.kegg.jp/) (17) and HMDB (http://www.hmdb.ca/) according to Tao et al. (18); then, the annotated metabolites were mapped to the KEGG pathway database (http://www.kegg.jp/kegg/pathway.html).

### 2.4 Statistical analysis

SPSS 21.0 software (IBM, Armonk, NY, USA) was used for statistical analysis of the final experimental data. GraphPad Prism 8.0 (La Jolla, CA, USA) was used to plot the histograms of the expression of seven key metabolites with mean ± standard error of the mean (SEM). Bioinformatics-related widely untargeted metabolomic analysis was performed using the OmicStudio tools (https://www.omicstudio.cn/tool) (accessed on 3 October 2022).

## 3 Results

### 3.1 Metabolic profile of supraspinatus tendon

**Figure 2:**
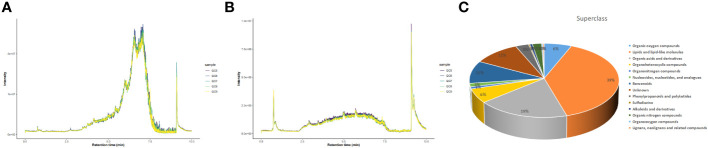
Total ion chromatograms of LC-MS data from metabolite profiles of QC samples detected in the POS ion mode **(A)** and NEG ion mode **(B)** X-axis represents retention time, and Y-axis represents total ion chromatogram in MS. **(C)** Superclass entries for all identified metabolites.

Total ion chromatograms (TIC) of QC samples tested in positive (POS) ion mode (**Figure 2A**) and negative (NEG) ion mode (**Figure 2B**) demonstrated the repeatability and reliability of the data. A total of 335 metabolites were detected in supraspinatus tendon samples, accounting for only 3.77% of the total metabolites, as seen by the results of untargeted metabolomics analysis. A total of 201 POS-mode metabolites (**Supplementary Table 1**) and 134 NEG-mode metabolites (**Supplementary Table 2**) were tentatively identified, which could be annotated into 14 classes (**Figure 2C**), mainly including lipids and lipid-like molecules (39.1%), organic acids and derivatives (18.81%), and benzenes (9.85%).

### 3.2 Principal component analysis (PCA) and partial least squares-discriminant analysis (PLS-DA)

**Figure 3:**
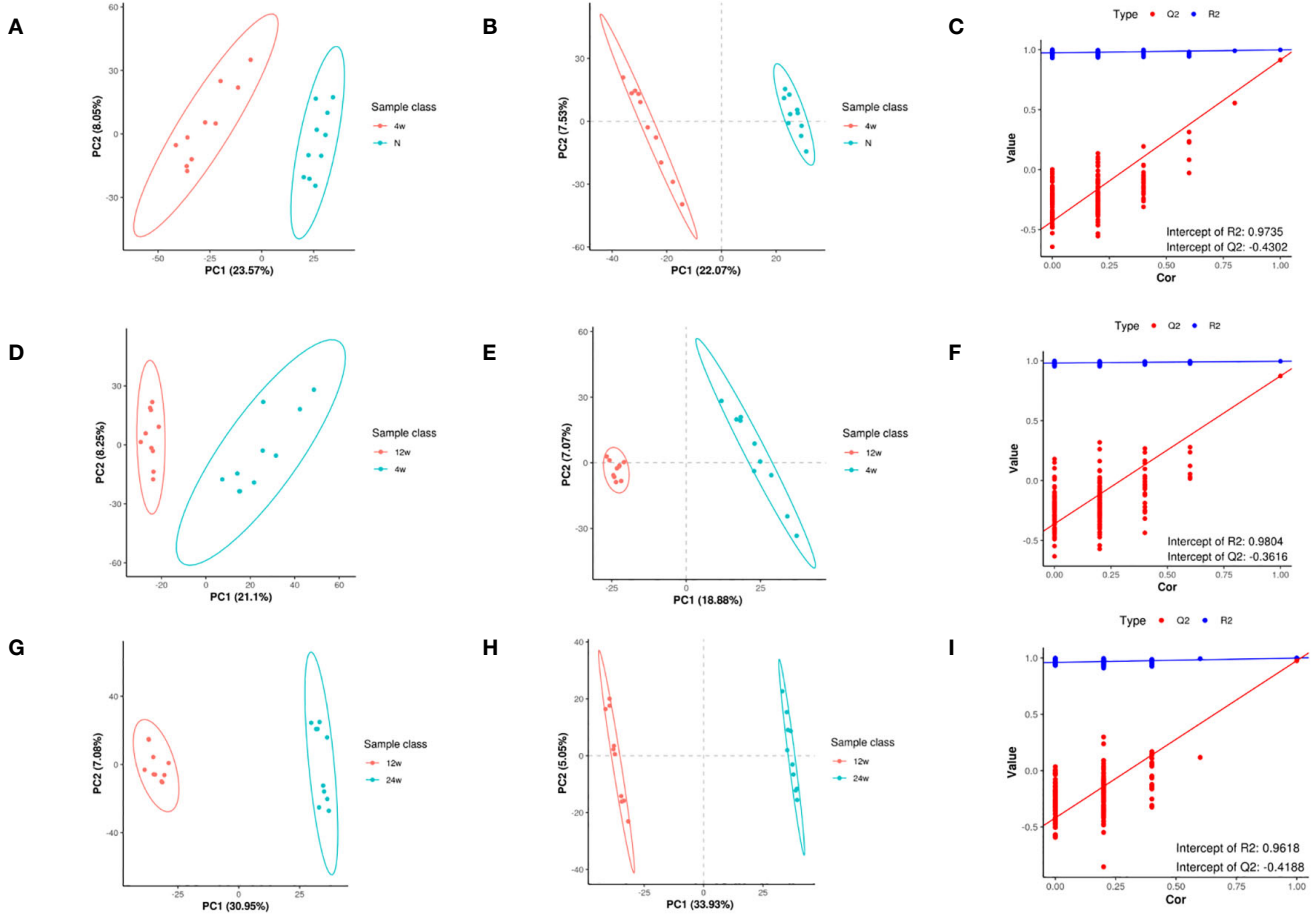
Metabolomic changes in rat supraspinatus tendon at different time points after diabetes induction compared to the normal group. PCA analysis **(A)**, PLS-DA **(B)**, and permutation analysis **(C)** in the T2DM-4w group vs. Normal group. PCA analysis **(D)**, PLS-DA **(E)**, and permutation analysis **(F)** in the T2DM-12w group vs. T2DM-4w group. PCA analysis **(G)**, PLS-DA **(H)**, and permutation analysis **(I)** in the T2DM-24w group vs. T2DM-12w group.

The PCA of metabolites (**Figures 3A, D, G**) showed the degree of separation between tendon tissues from different courses of diabetes, indicating that various stages of diabetes had diverse effects on tendons, indicating differences in metabolites between the two groups; also, the analytical method was reproducible. In order to maximize the differences between groups, PLS-DA provided a multivariate statistical analysis method with supervised pattern recognition, revealing distinct differences between groups and better clustering of sample points within groups (**Figures 3B, E, H**). PLS-DA model could overfit in processing high-dimensional data, and to prevent model overfitting, the permutation tests of PLS-DA model were conducted. Q2<0 in the permutation tests diagram (**Figures 3C, F, I**) indicates that there is no overfitting of the model, and the differential metabolite analysis is more accurate. In addition, the data in the blue line (R2) were higher than those in the red line (Q2), indicating that the model established by the experiment had not undergone overfitting, indicating its validity that could be analyzed further.

### 3.3 DEMs and their KEGG enrichment analysis

**Figure 4:**
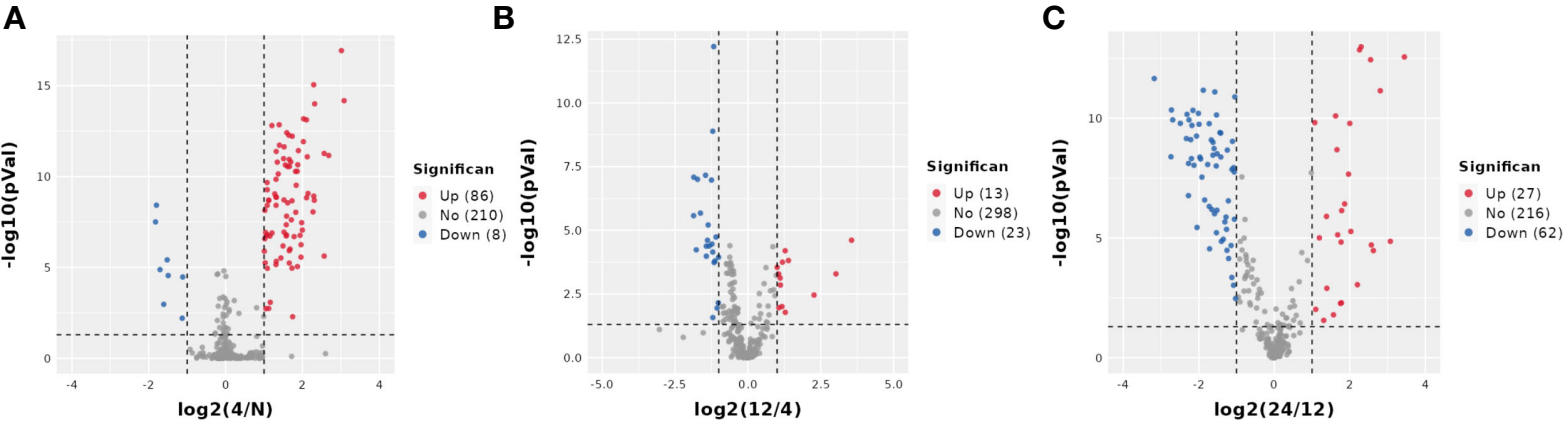
Volcano plot of DEMS between three comparison groups. Each dot represents one metabolite Red dots represent the significantly upregulated metabolites, and blue dots represent the significantly downregulated metabolites Gray dots represent no significant DEMS. **(A)** T2DM-4w group vs. Normal group: **(B)** T2DM-12w group vs. T2DM-4w group: **(C)** T2DM-24w group vs. T2DM-12w group.

**Figure 5:**
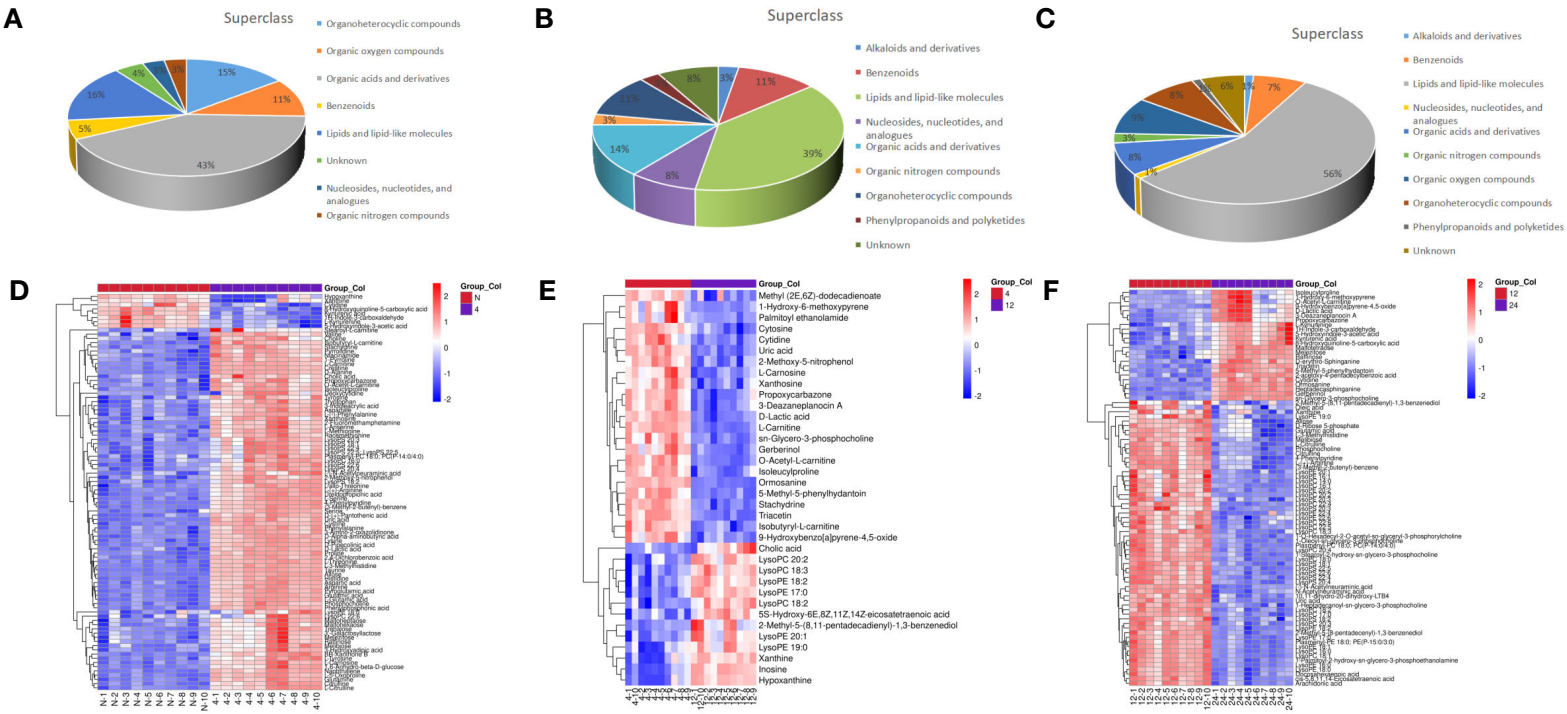
Classification of metabolites including T2DM-4w group vs. Normal group **(A)**, T2DM-12w group is, T2DM-4w group **(B)**, and T2DM-24w group vs T2DM-12w group **(C)**; Heatmap of annotated metabolites with increasing and decreasing trend, including T2DM-4w group vs. Normal group **(D)**, T2DM-12w group vs. T2DM-4w group **(E)**, and T2DM- 24w group vs. T2DM-12w group **(F)**. Each column represents a sample, and each metabolite is visualized in a row Red indicates a high abundance, and blue indicates a relatively low abundance of metabolites.

**Figure 6:**
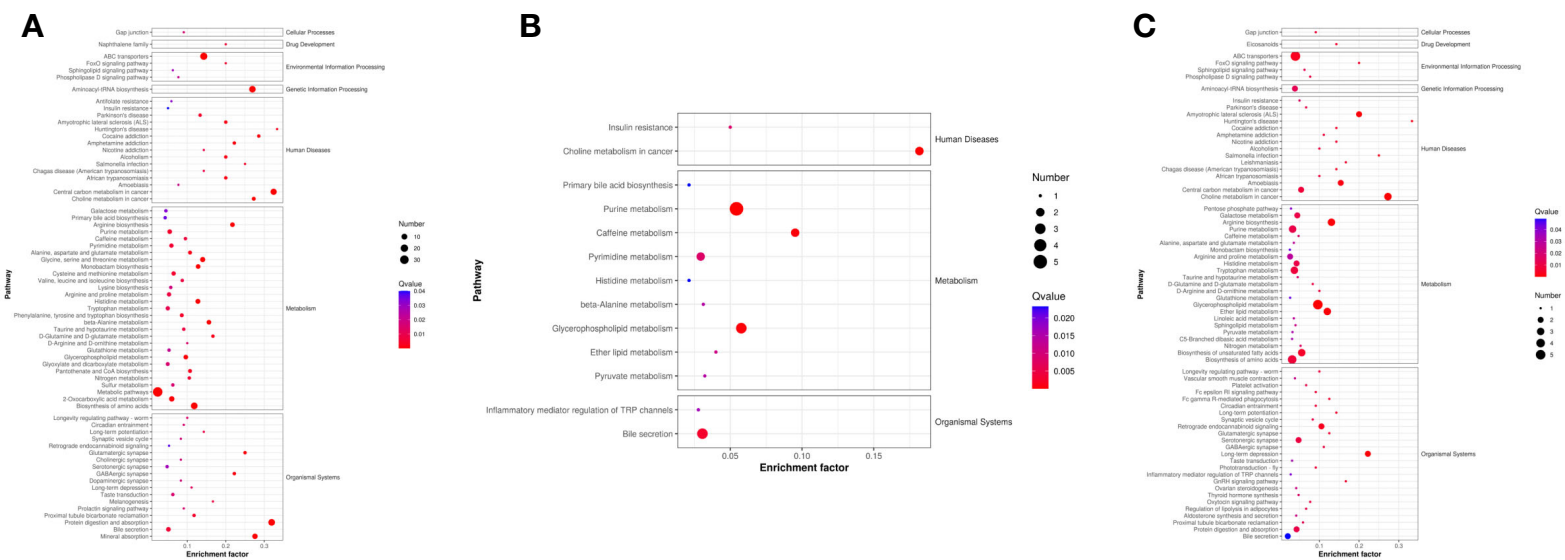
KEGG pathways that the distinguished metabolites participate in different comparison groups. **(A)** T2DM-4w group vs. Normal group. **(B)** T2DM-12w group vs. T2DM-4w group; **(C)** T2DM-24w group vs. T2DM-12w group. The color of the point represents the p-value. The smaller the value, the higher the reliability of the test and the greater the statistical significance. The size of the point represents the number of differential metabolites in the corresponding path. The larger the point, the more DEMs in the pathway.

Next, we analyzed the annotated metabolites in tendon tissue, and volcano plots were used to illustrate the distribution of DEMs at various time points of diabetes (**Figures 4A–C**). Red and blue circles in the volcano plot are up- and downregulated metabolites, and the data showed that the number of DEMs exhibits an increasing trend in a diabetes-duration manner (**Table 1**). A total of 94 DEMs were detected in the T2DM-4w group compared to NG (**Supplementary Table 3**), including 86 up- and 8 downregulated metabolites. The most affected metabolites were organic acids and derivatives (42.55%), lipid and lipid molecules (15.56%), and organic heterocyclic compounds (14.89%) (**Figure 5A**). DEMs were enriched into the KEGG database, following which, we detected the enrichment of 92 pathways (**Supplementary Table 6**, **Figure 6A**), of which the top three significantly enriched metabolic pathways were protein biosynthesis and absorption, aminoacyl-tRNA digestion, and ABC transporters. We also identified 36 DEMs in the T2DM-12w compared to the T2DM-4w group (**Supplementary Table 4**), including 13 up- and 23 downregulated metabolites. The most affected metabolites were lipids and lipid molecules (39%), organic acids and derivatives (14%), organic heterocyclic compounds (11%), and benzene (11%) (**Figure 5B**). DEMs were enriched in the KEGG database, and it was found that 17 pathways were enriched (**Supplementary Table 6**, **Figure 6B**), of which the top three significantly enriched metabolic pathways were purine metabolism, choline metabolism in cancer, and glycerophospholipid metabolism. A total of 86 DEMs were found in T2DM-24w compared to the T2DM-12w group (**Supplementary Table 5**), including 24 up- and 62 downregulated metabolites. The most affected metabolites were lipids and lipid molecules (56%), organo-oxygenated compounds (9%), organic acids and derivatives (8%), and organic heterocyclic compounds (8%) (**Figure 5C**). DEMs were enriched in the KEGG database, and it was found that 86 pathways were enriched (**Supplementary Table 6**, **Figure 6C**), of which the top three significantly enriched metabolic pathways were choline metabolism in cancer, glycerophospholipid metabolism, and arginine biosynthesis. DEMs from the three comparison groups could be aggregated by hierarchical cluster analysis (**Figures 5D–F**), and regions of different colors represent cluster grouping to visualize the variations in metabolites between groups by color gradients.

**Table 1:** Changes in differential metabolites in the three comparison groups.

Comparison	All	Up	Done	NEG	POS
T2DM-4w vs NG	94	86	8	40	54
T2DM-12w vs T2DM-4w	36	13	23	15	21
T2DM-24w vs T2DM-12w	86	24	62	46	40

### 3.4 Metabolomic alterations associated with the progression of diabetic tendinopathy

**Figure 7:**
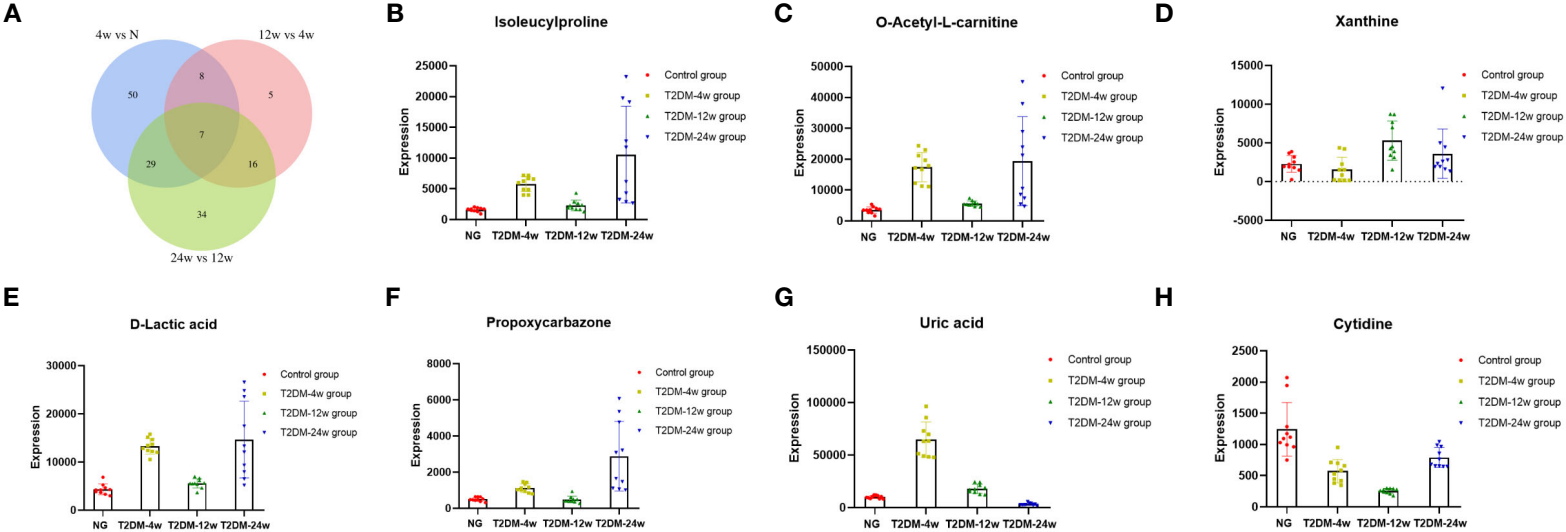
**(A)** The Venn diagram compares the number of different metabolites among all three comparison groups, including the paired comparison groups of T2DM-4w group vs. Normal group, T2DM-12w group vs. T2DM-4w group, and T2DM-24w group vs. T2DM-12w group. The different expression levels of isoleucylproline **(B)**, O-acetyl-L-carnitine **(C)**, xanthine **(D)**, D-lactic acid **(E)**, propoxycarbazone **(F)**, uric acid **(G)**, and cytidine **(H)** between Normal group, T2DM-4w group, T2DM-8w group, and T2DM-12w group. Adjusted p-value < 0.05 were selected as DE features.

**Figure 8:**
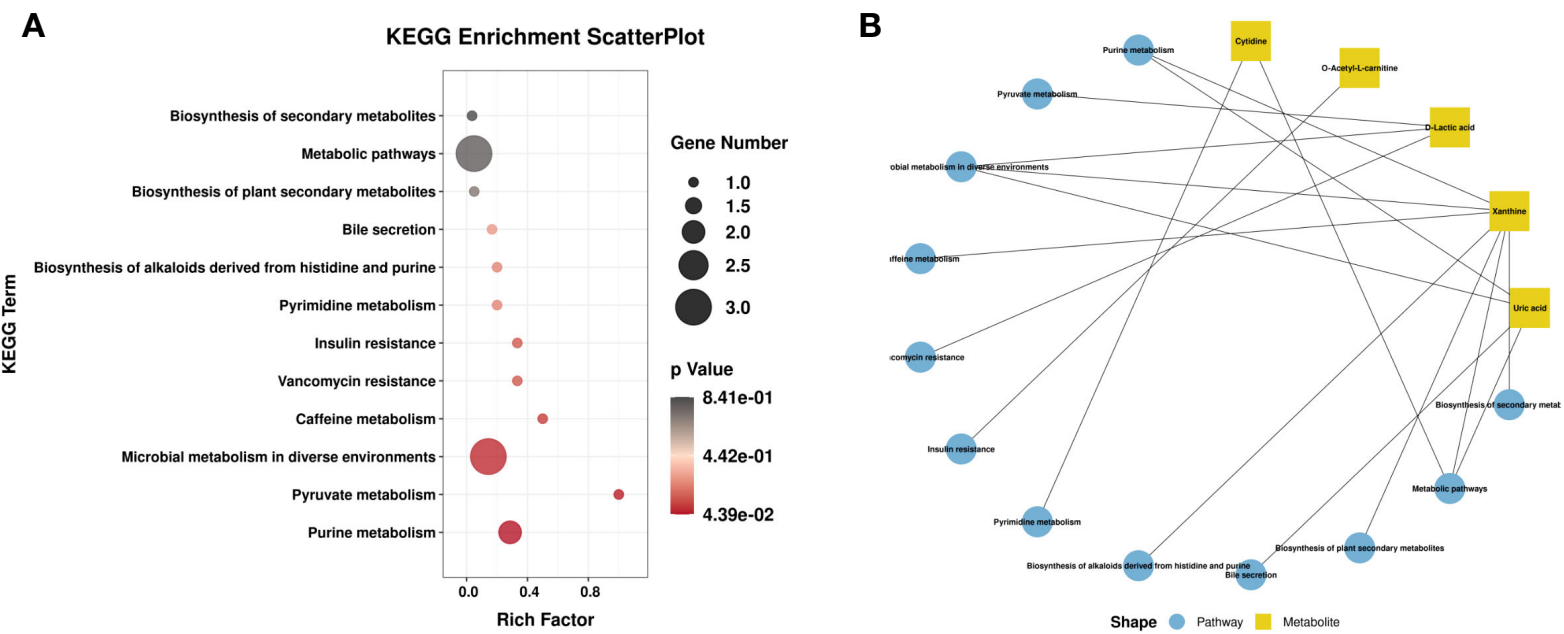
**(A)** Scatter plot of seven critical metabolites with the most significant enrichment. p-value is plotted on a color map. **(B)** Typical KEGG pathway vs. metabolite network graphs.

Venn diagram (**Figure 7A**) shows the DEMs co-existing in the three comparison groups. We found that seven selected metabolites were common DEMs in all comparison groups (**Table 2**), including D-lactic acid, xanthine, O-acetyl-L-carnitine, isoleucylproline, propoxyazone, uric acid, and cytidine, all of which were the first biomarkers identified in the supraspinatus tendon with the progression of diabetes (**Figures 7B–H**). Seven DEMs were subjected to KEGG enrichment analysis, and the results showed that a total of 12 metabolic pathways were enriched (**Table 3**), of which purine metabolic pathways were significantly enriched (p < 0.05), suggesting that purine metabolism is crucial for the progression of diabetic tendinopathy (**Figure 8A**). The results of the network diagram of DEM correlations in KEGG-enriched pathways (**Figure 8B**) revealed that uric acid and xanthine play a role in several metabolic pathways, and these two biomarkers may be closely associated with the development of diabetic tendinopathy.

**Table 2:** List of basic information on seven key metabolites associated with the progression of diabetic tendinopathy.

Compound name	m/z ^a^	rt^b^ (min)	Mode	KEGG ID	Trend		
					4w vs NG	12w vs 4w	24w vs 12w
Xanthine	151.0258883	1.497016667	POS	C00385	down	up	down
Uric acid	167.0210255	0.977166667	NEG	C00366	up	down	down
O-Acetyl-L-carnitine	204.1221238	0.963716667	POS	C02571	up	down	up
Propoxycarbazone	443.0774954	0.759583333	NEG	–	up	down	up
D-Lactic acid	89.0240203	1.316475	NEG	C00256	up	down	up
Isoleucylproline	229.1532649	0.972533333	POS	–	up	down	up
Cytidine	266.0732398	0.95165	POS	C00475	down	down	up

^a^mass to charge ratio of the features; ^b^retention time of the features.

**Table 3:** List of pathways enriched for seven key metabolites.

Pathway ID	Pathway Description	Matching IDs	Metabolite	p-value
Map00230	Purine metabolism	C00366;C00385	Uric acid, Xanthine	0.04
Map00620	Pyruvate metabolism	C00256	D-Lactic acid	0.05
Map01120	Microbial metabolism in diverse environments	C00366;C00256;C00385	Uric acid, D-Lactic acid, Xanthine	0.07
Map00232	Caffeine metabolism	C00385	Xanthine	0.11
Map01502	Vancomycin resistance	C00256	D-Lactic acid	0.15
Map04931	Insulin resistance	C02571	O-Acetyl-L-carnitine	0.15
Map00240	Pyrimidine metabolism	C00475	Cytidine	0.25
Map01065	Biosynthesis of alkaloids derived from histidine and purine	C00385	Xanthine	0.25
Map04976	Bile secretion	C00366	Uric acid	0.29
Map01060	Biosynthesis of plant secondary metabolites	C00385	Xanthine	0.71
Map01100	Metabolic pathways	C00366;C00475;C00385	Uric acid, Xanthine, Cytidine	0.80
Map01110	Biosynthesis of secondary metabolites	C00385	Xanthine	0.84

## 4 Discussion

Metabolomics is widely used in many fields and plays a critical role in the study of mechanisms involved in diabetes and its complications and the establishment of predictive models (19, 20). Metabolomics techniques involve the identification and characterization of small molecules; the commonly used analytical techniques include nuclear magnetic resonance (NMR), gas chromatography-mass spectrometry (GC-MS), and liquid chromatography-mass spectrometry (LC-MS) (21). Non-targeted metabolomics studies analyze endogenous small molecule metabolites in the body without bias, providing information on thousands of compounds detected in samples and discovering the correlation between metabolites and physiological and pathological changes (22). Because each individual’s metabolic profile is unique, fluctuations and differences in metabolite levels directly reflect the mechanisms underlying disease development and progression (23). Over the past decade, many studies have provided information regarding the mechanisms by which diabetes leads to the continuous progression and deterioration of the kidney (8), microvasculature (9), and retinal diseases (10) through untargeted metabolomics techniques. However, the correlation between metabolite profiles and the severity of diabetic tendinopathy has not yet been investigated.

This is the first study to establish a list of biomolecules that may be involved in the dynamic changes of the supraspinatus tendon of the rotator cuff with the progression of diabetes using mass spectrometry-based untargeted metabolomics techniques. In addition, we identified seven key metabolites that may be associated with the progression of rotator cuff tendinopathy, especially uric acid and xanthine play a role in metabolic pathways which might be closely related to the development of diabetic tendinopathy.

The comparison of time-dependent metabolic trajectories between diabetic and control groups provided information about metabolites that may be involved in the development of diabetic tendinopathy. In the present study, 12 pathways, such as purine, pyruvate, and microbial metabolisms in different environments, were likely to be associated with the development of diabetic tendinopathy, and one of the most significantly changed (p < 0.05) pathways was the purine metabolism pathway. Purines are critical components of the cellular energy system and are also critical components of pyrimidine, RNA, and DNA production (24). To the best of our knowledge, this is the first study reporting that diabetes alters purine metabolism in tendon samples. In addition, xanthine was first decreased at week 4 after induction of diabetes compared to the normal group, but significantly increased in tendons from week 4 to week 8 of diabetes and decreased at week 12, but the results were still higher than those in the normal group. Previous studies have also shown that the purine metabolic pathway is associated with gestational diabetes (25), and xanthine is significantly increased in the urine metabolome of subjects with gestational diabetes (26). A total of 17 metabolites were identified between type 2 diabetes and diabetic nephropathy; among these, purine metabolism is mainly involved in this disease (27). In addition, the pathophysiology of a high-sugar diet is associated with the dysregulation of purine metabolism (28). Another study recorded serum metabolomics data from 650 healthy people, showing that consumption of sugar-rich foods is closely related to elevated circulating purine levels. This finding suggested that dietary sugar affects human health through the dysregulation of purine metabolism (29).

The optimal concentrations of uric acid are essential for the normal functioning of the body (30). Compared to the control group, uric acid levels in the supraspinatus tendon of rats with diabetes for 4 weeks increased significantly and decreased gradually during the period from weeks 4–24 of diabetes. The accelerated accumulation of uric acid in the initial period after diabetes induction might contribute to the development and progression of diabetic complications. The precursor of uric acid is xanthine, which is further oxidized to uric acid by xanthine oxidase (31). Cytosol contains about 4 mg/mL of uric acid, which increases significantly after nucleic acid degradation in injured cells (32). In addition, high levels of serum uric acid levels are associated with glucose metabolism disorders (33). In a previous study, uric acid was increased in meconium or urine of newborns from mothers diagnosed with gestational diabetes (34). Elevated plasma/serum uric acid is associated with an increased risk of insulin resistance (35, 36), and pathogenic mechanisms may be related to the inhibition of insulin signaling and AMPK activity (37, 38).

However, uric acid, as a natural antioxidant *in vivo*, can scavenge toxic free radicals produced during physiological and pathological processes and plays a critical role in anti-oxidative stress, neuroprotection, and anti-inflammation (39). In humans, about 50% of the plasma antioxidant capacity is obtained from uric acid (40, 41). Uric acid acts as an antioxidant and can scavenge excessive reactive oxygen species (ROS) and peroxynitrite from the body (42). High levels of uric acid are detected in the cytoplasm of normal human and mammalian cells, which is also closely related to its antioxidant effects (43–45). In addition, uric acid plays a role in tissue repair, and the related mechanisms may be associated with initiating the inflammatory process and mobilizing progenitor endothelial cells (46). Uric acid also has a role in the prevention of disease; for example, peroxides and ROS can be blocked by high uric acid levels, and hence, the probability of multiple sclerosis (MS) is greatly reduced in patients with gout (47). Therefore, we hypothesized that the decrease in uric acid levels in the supraspinatus tendon from week 4–24 after diabetes induction might further aggravate oxidative stress and inflammatory response in the supraspinatus tendon, leading to the progression of tendinopathy; however, the specific mechanism needs to be verified by additional studies in the future.

According to the current results, a series of amino acids was significantly reduced in T2DM-24w compared to the T2DM-12w group, including L-citrulline, L-(+)-arginine, citrulline, and glutamic acid, which are critical substances to maintain the normal function of the human body. The metabolic processes of the body are extremely important, and glutamic acid is the most abundant amino acid in the mammalian brain. It is mainly involved in the synthesis of protein peptides and fatty acids, together with glutamine, and regulates the ammonia levels in the body (48). In addition, glutamate is an acidic amino acid, and although it is not an essential amino acid for the human body, it participates in body metabolism as a carbon and nitrogen nutrient (49). The alterations in amino acid metabolism in the supraspinatus tendon of diabetic rats may contribute to many clinical changes, and amino acid metabolites may be potential biomarkers.

T2DM-12w showed a significant increase in LysoPE (18:2), LysoPE (17:0), LysoPE (19:0), LysoPE (20:1), LysoPC (18:2), LysoPC (18:3), and LysoPC (20:2) compared to the T2DM-4w group. Lyso-phosphatidylcholine is a phospholipid closely related to metabolic diseases, such as diabetes, dyslipidemia, and atherosclerosis, and plays a key role in the inflammatory response as the key metabolite of the lipid pro-inflammatory pathway (50). Accumulation of LysoPC induces apoptosis and leads to mitochondrial dysfunction (51). *In vitro* studies have shown that LysoPC elicits apoptosis when incubated with cultured hepatocytes (52). However, high phosphatidylcholine levels were associated with a low risk of type 2 diabetes (20). The key metabolic pathway associated with these metabolites is glycerophospholipid metabolism, a type of lipid metabolism, and abnormal lipid metabolism is directly associated with oxidative stress and inflammatory responses (53). Compared to the T2DM-12w group, some LysoPC decreased in the T2DM-24w group, although the related mechanism needs to be explored further. The current results suggested that abnormal glycerophospholipid metabolism may be one of the metabolic pathways involved in the progression of diabetic tendinopathy.

As the first study to investigate the dynamic changes of metabolites in the supraspinatus tendon of the rotator cuff in diabetic rats using untargeted metabolomics techniques, the experimental results prompt the exploration of the mechanism of the development and progression of diabetic tendinopathy. Nevertheless, the present study has some limitations. First, we identified several metabolites that continue to change with the duration of diabetes as no similar studies have previously corroborated these findings; hence, it is difficult to understand and interpret these results in the development of diabetic tendinopathy. While the identified metabolites are promising as potential biomarkers, further validation in larger cohorts of both animals and humans is needed to confirm their specificity and relevance to tendinopathy. Second, While the study identifies key metabolites and affected pathways, it may not provide a complete mechanistic understanding of how these metabolites contribute to diabetes-induced tendinopathy. Further research is required to explore the causal relationships. Third, the development of diabetic tendinopathy can be divided into several stages, such as “early,” “middle,” and “late.” These experimental groupings also attempted to investigate the potential differences in metabolites in these three stages; however, due to the lack of stage information on the pathogenesis of tendinopathy in diabetic rats, we were unable to completely mimic the pathogenesis of diabetic tendinopathy in this study. Therefore, the present results do not facilitate definitive and straightforward conclusions unless validated in subsequent diverse samples. Fourth, because the metabolism of different individuals is different and the sample size is limited, it is difficult to include relevant experiments in three or more replicates. Therefore, future studies need to replicate the experiment in parallel control with a larger sample cohort. Finally, untargeted metabolomics, as a broad and target-less detection modality, yields results that do not allow quantitative analysis of metabolites; therefore, combining various platforms and multi-omics in further studies is crucial.

## 5 Conclusions

In this study, we established for the first time a biomolecule list of dynamic changes in rat supraspinatus tendon with the progression of diabetes using mass spectrometry-based untargeted metabolomics techniques. Moreover, seven key metabolites detected in the supraspinatus tendon continue to change with diabetes progression. Especially the discovery of uric acid and xanthine may provide novel ideas for exploring the mechanisms of diabetic tendinopathy progression.

## Data availability statement

The original contributions presented in the study are included in the article/**Supplementary Material**. Further inquiries can be directed to the corresponding authors.

## Ethics statement

The animal study was approved by the Ethics Committee of Experimental Animals of the Affiliated Hospital of Qingdao University (No. 20220505SD8020221210126). The study was conducted in accordance with the local legislation and institutional requirements.

## Author contributions

KX: Writing – original draft. LZ: Data curation, Writing – original draft. TW: Methodology, Writing – original draft. ZR: Data curation, Project administration, Writing – original draft. TY: Supervision, Writing – review & editing. YZ: Supervision, Writing – review & editing. XZ: Writing – review & editing.

## Supplementary material

Supplementary Table 1 A total of 201 metabolites were identified from 5758 metabolite features extracted from the raw data acquired in positive-ionization modes by untargeted metabolomics analysis.

Supplementary Table 2 A total of 134 metabolites were identified from 3118 metabolite features extracted from the raw data acquired in negative-ionization modes.

Supplementary Table 3 A total of 94 differential metabolites (86 up-regulated and 8 down-regulated) were identified in the T2DM-4w group compared with the normal group.

Supplementary Table 4 A total of 36 differential metabolites (13 up-regulated and 23 down-regulated) were identified in the T2DM-12w group compared with the T2DM-4w group.

Supplementary Table 5 A total of 86 differential metabolites (24 up-regulated and 62 down-regulated) were identified in the T2DM-24w group compared with the T2DM-12w group.

Supplementary Table 6 KEGG pathways, in which differentially expressed metabolites from the three comparison groups are involved. Dots are colored to represent P-values. The smaller the value, the greater the reliability of the test and the greater the statistical significance. The size of the dots represents the number of differentially expressed metabolites in the corresponding path. The larger the point, the more differentially expressed metabolites in the pathway.
